# A Knowledge Graph Entity Disambiguation Method Based on Entity-Relationship Embedding and Graph Structure Embedding

**DOI:** 10.1155/2021/2878189

**Published:** 2021-09-23

**Authors:** Jiangtao Ma, Duanyang Li, Yonggang Chen, Yaqiong Qiao, Haodong Zhu, Xuncai Zhang

**Affiliations:** ^1^College of Computer and Communication Engineering, Zhengzhou University of Light Industry, Zhengzhou 450002, China; ^2^The State Information Center, Beijing 100045, China; ^3^College of Information Engineering, North China University of Water Resources and Electric Power, Zhengzhou 450046, China; ^4^College of Electrical and Information Engineering, Zhengzhou University of Light Industry, Zhengzhou 450002, China

## Abstract

The purpose of knowledge graph entity disambiguation is to match the ambiguous entities to the corresponding entities in the knowledge graph. Current entity ambiguity elimination methods usually use the context information of the entity and its attributes to obtain the mention embedding vector, compare it with the candidate entity embedding vector for similarity, and perform entity matching through the similarity. The disadvantage of this type of method is that it ignores the structural characteristics of the knowledge graph where the entity is located, that is, the connection between the entity and the entity, and therefore cannot obtain the global semantic features of the entity. To improve the Precision and Recall of entity disambiguation problems, we propose the EDEGE (Entity Disambiguation based on Entity and Graph Embedding) method, which utilizes the semantic embedding vector of entity relationship and the embedding vector of subgraph structure feature. EDEGE first trains the semantic vector of the entity relationship, then trains the graph structure vector of the subgraph where the entity is located, and balances the weights of these two vectors through the entity similarity function. Finally, the balanced vector is input into the graph neural network, and the matching between the entities is output to achieve entity disambiguation. Extensive experimental results proved the effectiveness of the proposed method. Among them, on the ACE2004 data set, the Precision, Recall, and *F*1 values of EDEGE are 9.2%, 7%, and 11.2% higher than baseline methods.

## 1. Introduction

Knowledge graph entity disambiguation is to match entity mentions in facts to corresponding entities in a given knowledge graph. Knowledge graph entity disambiguation is a primary technique in the course of relationship extraction and knowledge graph fusion. It aims to resolve the widespread entity ambiguity problem in the process of knowledge graph construction. It is widely used in knowledge graph reasoning [1], information retrieval [2], intelligent recommendation [3], and question answering systems [4].

Knowledge graph entity disambiguation is unlike named entity recognition. The former is to disambiguate entities with the equal name in the knowledge graph; that is, an entity has multiple interpretations, and inconsistent entities need to be filtered based on semantic similarity; the latter is to identify the entity from the text, but it is unknown what this entity refers to. The current entity disambiguation methods typically consider only use of translation-based models to obtain the mentioned entity embedding vector [5–7], compare it with the candidate entity embedding vector, and then consider the global consistency and use of statistical model to remove ambiguous entity. The translation-based models employ a representation learning method to capture the relation feature between entities. The weakness of these models is that the global structure feature between entities is not encoded in the embedding presentation. Another line of work utilizes neural networks to do entity disambiguation in an end-to-end way [8, 9] (joint entity and relation extraction model based on rich semantics), which utilizes entity-relation embedding and a differentiable joint inference method for entity disambiguation. Neural network-based entity disambiguation methods cannot capture the global structure feature of the knowledge graph and have poor explanation.

**Figure 1:**
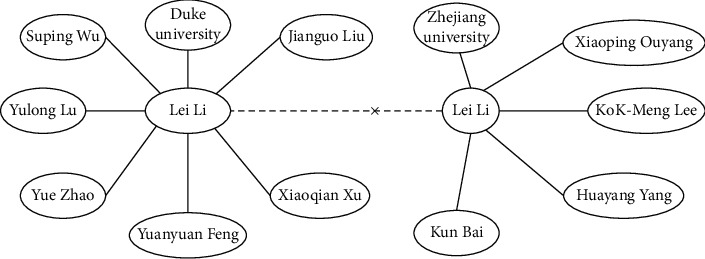
Example of graph of knowledge graph entity disambiguation.

Figure 1 shows an example of knowledge graph entity disambiguation. In the DBLP [10] database, there are 38 authors named Lei Li. The latest method based on the statistical model cannot map the mentioned “Lei Li” to the correct entity “Lei Li” and cannot accurately disambiguate these ambiguous entities. The reason may be that collective entity disambiguation using CRF is not sufficient to capture global structural information. Nevertheless, if we construct an entity-relationship graph to encode the global structural relationship between ambiguous entities in the facts, we find the structure information of “Lei Li” from Duke University and the graph structure of “Lei Li” from Zhejiang University is different. Thus, the graph structure information can be used to capture the global characteristics of the entity in the knowledge graph. The subgraphs around “Lei Li” are different, and the “Lei Li” in the left graph is different from “Lei Li” in the right graph. There should be a dotted line between them since they are not the same entities.

Entity relation represents the semantic relationship between entities, and the entity's neighbor graph contains the structure feature between entities. Relational graph convolutional network has a good ability to model relational data. Inspired by the above ideas, we propose an entity disambiguation model based on entity-relationship embedding and entity subgraph structure embedding (EDEGE). First, as shown in Figure 1, an entity-relation graph is built based on facts to simulate the global structural relationship between ambiguous entities in a batch of facts. The entity-relation graph is built according to the relation between head entity and tail entity; the facts who share the same head entity or tail entity are linked to the entity-relation graph. The mentioned entities may correspond to two or more entities. The entities in the figure are selected from the head and tail entities in the facts. Then, the graph neural network is utilized to generate entity-relationship embedding with graph structure characteristics. The semantic feature is propagated along with the entity-relationship graph, and the global structure characteristics between these entity relationships are encoded. Thirdly, EDEGE concatenated the entity-relation embedding and entity's subgraph embedding, which are used as the input of relational graph neural network to disambiguate the ambiguous entities in an end-to-end way.

In our model, the richly structured entity embedding vector generated by graph neural network can better eliminate the ambiguity between candidate entities and increase entity disambiguation's accuracy. The entity-relation graph is built for every batch of facts to be disambiguated, so the specific structure in the invisible facts could be converted to the entity-relation embedding vector so that our model is easy to adapt to new facts in the testing phase, which can disambiguate entities during the construction of knowledge graph and newly added entities during the dynamic update of the knowledge graph. All in all, our contribution has the following three aspects:

1. As far as we know, this paper first proposed a relational graph convolutional network to do the entity disambiguation in an end-to-end way. The entity-relation embedding is generated through entity matrix and relation matrix; it represents the semantic relationship between entities. The entity relationship contains the global structure and semantic relations between entities and relations and uses a graph neural network to encode on the graph global entity embedding vector to improve the precision of entity disambiguation.
2. We utilize the entity's adjacent subgraph feature to represent the entity's graph features. The entity's adjacent neighbor subgraph embedding is trained through a relational graph convolutional network. The entity-relation embedding and entity's subgraph embedding are concatenated, which is input into a relational graph convolutional network to do entity disambiguation in an end-to-end way, thereby further improving the reasoning recall rate of entity disambiguation, and the entity disambiguation method has better interpretability.
3. Extensive experimental results on the public data sets proved the validity of EDEGE compared with baseline methods. Take ACE2004 data set, for example. The Precision, Recall, and *F*1-measure values of EDEGE are 9.2%, 7%, and 11.2% higher than those of the second best method GNED.

The remainder of this paper is arranged as follows.  Section 2 introduces the related work of the knowledge graph entity disambiguation problem.  Section 3 describes the knowledge graph entity disambiguation problem in detail.  Section 4 introduces the proposed graph neural network entity disambiguation model EDEGE.  Section 5 shows the experimental results on public data sets. The last section comes to conclusions and gives the next research direction.

## 2. Related Work

Entity disambiguation methods are categorized into two classes. One is entity features-based entity disambiguation methods. This type of method disambiguates entities according to the semantic characteristics of the entity and relation, the context characteristics of the entity and relation, and the frequency characteristics of the entity's appearance. The other is the neural network-based entity disambiguation method. This type of method uses the graph structure features in the knowledge graph and utilizes the neural network model to perform end-to-end entity disambiguation.

### 2.1. Entity Features-Based Entity Disambiguation Method

The disambiguation method based on entity features performs entity disambiguation according to features and feature combinations. An entity similarity model is proposed to measure the difference between ambiguous entities. The named entity disambiguation system DBpedia Spotlight [11] mainly relies on entity context similarity measures for disambiguation. Adjali et al. [12] used entity semantic similarity, context similarity, and mention probability for entity disambiguation. Hoffart et al. [13] fused features that mentioned probability, entity similarity, and similarity of candidate entities based on graph links and used linear models to fuse these features for entity disambiguation. MCKR [14] uses the multilayer perceptron to extract interaction features of missing data and observational data.

By fusing entities, names, texts, and Wikipedia information in a probability model from different data sources, Barrena et al. [15] found that these features have obvious complementary effects in entity disambiguation. Houlsby and Ciaramita [16] are proposing solutions using a generative probability model with Latent Dirichlet Allocation model, this scheme constructs a topic model based on a specific knowledge base, where every topic corresponds to a Wikipedia page. Ganea et al. [17] offered a probability method PBoH that does not depend on any data set for joint entity disambiguation. PBoH relies on the statistical data of hyperlinks on Wikipedia on the cooccurrence entity to perform entity disambiguation. These statistics describe the cooccurrence probability of mention and entity pairs. PBoH considers every anchor word as a mentioned entity, the wikitext referred to is used as the reference data for the entity tag. Zwicklbauer et al. [18] offered an entity semantic embedding representation model for entity disambiguation. They used the Word2Vec [19] method to embed the entity and used the random walk method on the RDF graph to construct the entity sequence. Whether it is based on clustering or based on entity linking, the calculation of the similarity between entity and entity, entity and text, and text and text is the core issue in entity disambiguation. These calculation methods mainly use natural language processing techniques to extract entity's features. Although these methods have achieved good performance, the feature scalability is poor, the representation ability is insufficient, and it is easy to cause error propagation in entity disambiguation.

### 2.2. Neural Network-Based Entity Disambiguation Method

The neural network-based entity disambiguation method uses an end-to-end mechanism to increase the accuracy of entity disambiguation. Besides the entity-relation feature, researchers have integrated the graph structure features of the knowledge graph to further increase the effect of entity disambiguation. RS-Joint [20] integrates convolutional and recurrent neural networks to disambiguate entities and extract relations together. It can acquire rich semantics and utilizes the full advantage of the associated information between entities and relations need not external features. Guo and Barbosa [21] achieved the purpose of entity disambiguation by estimating the Topic-sensitive PageRank value of the candidate entity [22] and combining the random walk method on the knowledge graph to perform entity disambiguation. Alhelbawy and Gaizauskas [23] used a graph-based method to perform joint entity disambiguation. This method represents all entities in the text as nodes in the graph, then sorts them according to the PageRank value of the nodes, and performs entity disambiguation according to the size of the value. Singh et al. [24] utilized a graph model for entity disambiguation between documents. DoSeR [25] designed a collective disambiguation method utilizing the Personalized PageRank value on the knowledge graph of mentioned entities, which relies on the collective link algorithm for entity disambiguation. Recently, researchers have tried to use deep learning methods for disambiguation and achieved good results. Ganea and Hofmann [26] used the entity embedding in the knowledge graph, applied the attention-based method to obtain the embedding vector, and considered the coherence between entities for joint disambiguation. Different from relying on supervised or heuristic methods to predict entity relationships, [27] treated the relationships as hidden variables in the neural entity link model to achieve entity disambiguation with an end-to-end mechanism. DeepType [28] solves the problem of entity disambiguation by combining a symbolic feature and a typical feature into the inference of neural network. Researchers conceived a type model and utilized it to limit the output of the network to adapt to the structure feature. They proposed a two-stage algorithm for entity disambiguation, first creating a type system and then using it to train a neural network.

GNED [9] uses a graph neural network model to solve entity disambiguation problems. GNED constructs a graph containing entity and mentioned word for every text to build the global semantic relation between ambiguous entities in the text. The graph neural network trains the entity word graph to get entity graph embedding that encodes the global semantics feature, and the embedding presentation is transferred to the statistical model to remove disambiguate entity. Although existing methods apply CRF to entity disambiguation based on overall consistency information, global structural information is not fully utilized. Since the statistical model combines the global consistency of the actual entities through the paired potential function, the effect is quite limited. The statistical model cannot combine the global structural relationship between candidate entities and related words, and these entities can be utilized as a semantic link between entities to eliminate the ambiguity of candidate entities. Therefore, statistical models cannot acquire global consistency feature completely. The structure feature of entity is neglected, which is very important in the analysis knowledge graph's feature and the relationship between entities. Node2vec [29] points out that a node's graph feature can be represented by its neighbors.

Reference [8] proposed a deep neural network method NeuPL to compute the semantic similarity between entities. NeuPL is the first model of using a long short-term memory network to eliminate entity ambiguity. The limitation of the neural network-based method is that this method lacks good explanation.

Inspired by the above methods, this paper comprehensively considers the semantic features of the entity-relation and the subgraph structure of the knowledge graph and uses these features as the input of the graph neural network to disambiguate the entity in the knowledge graph. Thus, we can utilize the global semantic of entity-relation and global structure feature between entities and can provide a good explanation for entity disambiguation.

## 3. Problem Statement

The ambiguity of named entities means that one entity reference item can correspond to multiple real-world entities. For example, in the DBLP database, there are 37 authors named Lei Li, and we use Figure 1 to show the partnership graph of two of them. Determining the real-world entity pointed to by an entity referent is named entity disambiguation. Let *d* be a document, where all the named entities mentioned are marked by the entity disambiguation process, KG = (*H*, *R*, *T*) represents a knowledge graph, the nodes in *H* and *T* correspond to those entities in the actual world, *R* denotes the relationship between entities, (*h*, *r*, *t*) represents a fact in the knowledge graph, *h* ∈ *H*, *r* ∈ *R*, and *t* ∈ *T*. Entity disambiguation is to determine whether there is a conflict between entities when a new fact is added to the knowledge graph. If there is no conflict, add this fact to the knowledge graph. When there is a conflict, the conflicting entities are disambiguated through the disambiguation method.

**Definition 1 .**

Given a set of facts (*H*′, *R*′, *T*′) and knowledge graph KG = (*H*, *R*, *T*), then the entity disambiguation problem is to discover a match function Γ : *M*⟶*H* ∪ *T* ∪ {NEW}. Among them, *NEW* represents an entity that does not exist in KG (also called an entity outside KG).

To determine whether there is a conflict between two entities if entity's similarity is higher than a threshold value, they are considered to be similar entities and there is no conflict; otherwise, they are regarded as conflicting entities. The similarity sim(*h'*, *h*) between the entity *h*′ ∈ *H*′ in the new fact and the ambiguous entity in the entity *h* ∈ *H* in the knowledge graph is defined as

(1) $$\begin{array}{c} \left\{\text{sim}\left(h^{\prime },h\right)=\beta {\text{sim}}_{\text{KGE}}\left(h^{\prime },h\right)+\left(1-\beta \right){\text{sim}}_{\text{KGSE}}\left(h^{\prime },h\right),\right\} \end{array}$$

where sim_KGE_(*h*′, *h*) describes the similarity of the embedding vector of entity relationship and sim_KGSE_(*h*′, *h*) is the similarity of the knowledge graph structure corresponding to the entity. We use entity-relation similarity and graph structure-based similarity where the entity is situated to measure the similarity of two ambiguous entities and perform entity disambiguation based on the similarity function.

We use neighbor mention entity's feature vector and candidate entity's neighborhood structure to identity mention entity m_*i*_'s correct candidate entity *e*_1_^*i*^. Given each candidate entity *e*_*j*_^*i*^'s feature vector *f*^*ij*^ ∈ *R*^*d*0^ and subgraph representation *g*^*ij*^, where *e*_*j*_^*i*^ ∈ Φ(*m*_*i*_), Φ(*m*_*i*_) is the set of *m*_*i*_'s candidate entity, *g*^*ij*^ ∈ *R*^2*qn*^, R^2*qn*^ is the set of *g*^*ij*^'s adjacent subgraph, and *q* is the size of sliding window. We utilize them as the input of entity *m*_*i*_'s input: *f*=[*f*_*i*1_,…,*f*_*in*_]^*T*^ ∈ *R*^*n*×*d*0^; adjacent matrix $A={\left[{\hat{g}}_{1},\ldots ,{\hat{g}}_{n}\right]}^{T}\in R^{n\times \left(2qn+1\right)}$, where ${\hat{g}}^{j}={\left[g^{j},1\right]}^{T}\in R^{2qn+1}$ represent subgraph with self-connection. We normalize the sum of every row to 1 to avoid the different effect because of different data scale. Given *f* and $\tilde{A}$, the objective of entity disambiguation is to find the best assignment:

(2) $$\begin{array}{c} \Gamma^{*}\left(m_{i}\right)=\underset{\hat{y}}{\arg\max}P\left(\hat{y};f,\tilde{A},\omega \right), \end{array}$$

where $\hat{y}$ is the output of the candidate entity, *P*(·) is the probability function, and *ω* is the training parameter.

## 4. Proposed Method

**Figure 2:**
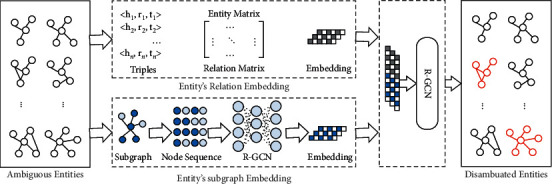
The framework of the proposed EDEGE method.

The entity graph embedding method builds semantic relationship*s* between neighbor entities. When new facts need to be added to the knowledge graph, the head entity and tail entity should be checked if they have ambiguity entities in the knowledge graph. If they have, we use EDEGE to disambiguate these entities. If not, the facts can be added to the knowledge graph. The framework of EDEGE is shown in Figure 2. Firstly, EDEGE utilizes a multilayer perceptron to get the entity-relation embedding vector. Then, EDEGE uses the entity's adjacent subgraph to get its structure embedding vector. Thirdly, the entity-relation embedding and entity's adjacent subgraph embedding are concatenated as an embedding with semantic and subgraph, which is as the input of relational graph neural network. Finally, EDEGE uses graph neural network to disambiguate entity in an end-to-end way. Taking Lei Li from Figure 1 as an example, firstly, the triples contain ambiguous entities Lei Li's relations are input in entity matrix and relation matrix to get the entity-relation embedding. At the same time, Lei Li's node sequence is generated from adjacent neighbors subgraph with a sample strategy. Then, the node sequence is input into a relational graph convolutional network to get the node's subgraph embedding. Thirdly, the entity-relation embedding and subgraph embedding are concatenated to be the input of relational graph convolutional network, which disambiguates the entities in an end-to-end way.

### 4.1. Entity-Relation Embedding

Given a set of tuples (*h*, *r*, *t*), entity *h* ∈ *H*, *t* ∈ *T*, and *r* ∈ *R*, EDEGE trains the embedding vector of entity and relation. TransE [30] is the first model to project the entity relation into low-dimension embedding and get good results on link prediction in a knowledge base. Inspired by TransE, we utilized the translation-based model to get the entity-relation embedding vector. The vector uses the median value of *N*^*k*^ (*k* is the hyperparameter of the proposed model and represents the number of context words around the entity) and uses the same letter to represent it. The basic idea of our model is that the edge labeled *r* corresponds to the embedded translation; that is, when (*h*, *r*, *t*) is true, *h* + *r* ≈ *t* is also true, and when (*h*, *r*, *t*) is not true, *h* + *r* and *t* have a big distance in similarity. Based on the framework of the energy model, the energy of the tuple is *d*(*h* + *r*, *t*), where *d* is a dissimilar measurement method, and we can use *L*_1 − norm_. To learn this embedding, we minimize the cost of the ranking principle with the training model:

(3) $$\begin{array}{c} L=\sum_{\left(h,r,t\right)\in S}\sum_{\left(h^{\prime },r,t^{\prime }\right)\in S{'}_{\left(h,r,t\right)}}{\left[r+d\left(h+r,t\right)-d\left(h^{\prime }+r,t^{\prime }\right)\right]}_{+}. \end{array}$$

Among them, [*x*]_+_ represents the positive part of *x*, and formula (4) restricts *S*_(*h*′, *r*, *t*)_′:

(4) $$\begin{array}{c} S_{\left(h^{\prime },r,t\right)}^{\prime }=\left\{\left(h^{\prime },r,t\right)|h^{\prime }\in E\right\}\cup \left\{\left(h,r,t^{\prime }\right)|t^{\prime }\in E\right\}. \end{array}$$

According to the wrong tuple set constructed by formula (3), the head entity or the tail entity is substituted by a randomly selected entity. The energy value of the loss function (1) to the training tuple is lower than the wrong tuple. Note that, for an entity, whether it is the head entity or the tail entity in the tuple, the embedding vector *V*_KGE_ is the same. *V*_KGE_ embedding for entities and relationships are initialized following a random process. The parameters are updated by using a gradient step with a learning rate. The training process is stopped based on its performance on a validation set.

### 4.2. Entity Graph Embedding

Besides the semantic features of entity relationships, the structural features between entities are also very important to identify the disambiguate entities. EDEGE utilizes the entity's relation to extract entity's adjacent subgraph feature, which is very useful to find the most coherent subset of the candidate entity. For every entity to be disambiguated, EDEGE extracts every entity's *e*_*∗*_^*i*^ subgraph feature g^*i*∗^, *e*_*∗*_^*i*^ ∈ Φ(*m*_*i*_), and *m*_*i*_ ∈ *M*. The subgraph *G*^*i∗*^=(*e*_*∗*_^*i*^ ∪ Φ(*N*(*m*_*i*_)), *R*^*i∗*^), where *R*^*i∗*^={*r*_*jk*_^*i∗*^*|e*_*k*_^*j*^ ∈ Φ(*m*_*j*_)}, *j* ∈ [*i* − *p*, *i* + *p*]\*i*}. EDEGE uses adjacent matrix-based vector to represent G^*i*∗^: *g*^*i∗*^=[*r*_*i∗i*−*p*,1_,…, *r*_*i∗i*+*p*,*n*_], *T* ∈ *R*^2*pn*^, where *n* is the number of candidate entities for mention entity, *p* is the size of the sliding window, and it represents the number of entity neighbors. Finally, for every candidate entity *e*_*j*_^*i*^, EDEGE concatenates its local feature and neighbor entity's coherent score as feature vector *f*^*ij*^ and constructs subgraph representation *g*^*ij*^ as the input of graph neural network.

The structural features of an entity node in the knowledge graph can be represented by its neighbor entity nodes. To use the relation feature of entities and their neighbor nodes in a knowledge graph, we take the structural feature learning in the knowledge graph as a maximum likelihood optimization problem. The knowledge graph needs a mapping function from the entity feature to prepare for the entity disambiguation tasks.

For the large knowledge graph, the computational cost is expensive, and EDEGE utilizes the negative sampling strategy for approximate calculation. For the model parameters of feature *f*, EDEGE utilizes the stochastic gradient descent strategy to optimize. Given a line of text, use a string of words as a sliding window to represent the neighbor characteristics of the words. However, the network characteristics of the knowledge graph cannot be solved by this linear method. To solve this problem, EDEGE utilizes a random walk process to sample multiple neighbor nodes of the entity node. Neighbor nodes are not limited to direct neighbors but can have different structures according to different sampling strategies. Here, the adjacent neighbor node is chosen to simulate the process of selecting neighbor nodes in the knowledge graph to get *V*_KGSE_.

### 4.3. Entity Disambiguation Based on Graph Neural Network

The final entity disambiguation is based on the similarity measurement of the entity embedding vector *V*_KGE_ and the entity structure embedding vector *V*_KGSE_. The splicing of vectors is the input of the graph neural network for entity disambiguation. The concat of *V*_KGE_ and *V*_KGSE_ is used as the input of graph convolutional network, which is an end-to-end model to do entity disambiguation through entity linking. The entity-relation-specific representation is compared with the complete relationship candidate set of each candidate entity, where each candidate relationship is also represented by its knowledge graph embedding. To match the relationship-specific problem representation with the candidate relationship of a given entity, we estimate the cosine similarity of the corresponding KG embedding and then rank all candidate relationships of entities that produce the entity-specific similarity according to the degree of similarity. To eliminate ambiguity, the entity-based similarity vector sim_KGE_ is passed to the gating mechanism:

(5) $$\begin{array}{c} g_{\text{amb}}=W_{g}V_{\text{KGE}}, \end{array}$$

where *W*_*g*_ ∈ *R*^*n*×1^ is to estimate whether there is more than one possible candidate in the entity candidate set based on the entity similarity. In addition, the structure vector *V*_KGSE_ based on the subgraph in which the entity is located should also be considered. This is by splicing the vectors of *V*_KGE_ and *V*_KGSE_ and predicting the final candidate entity through

(6) $$\begin{array}{c} y^{h}=\sigma \left(g_{\text{amb}}*\text{concat}\left(V_{\text{KGE}},V_{\text{KGSE}}\right)\left.+\left(1-g_{\text{amb}}\right)*V_{\text{KGE}}\right)\right). \end{array}$$

Note that *y*^*h*^ ∈ *R*^*n*^ is the number of ambiguous entities from which the entity with the highest probability can be selected. During the inference process, we perform additional steps to ensure that the entities and relationships predicted from the model form a pair in the knowledge graph. To achieve this goal, EDEGE selects the first few possible relationships from the relationship linker and selects the relationship with the highest probability as the relationship of the predicted entity.

EDEGE utilizes a graph convolutional network to extract the entity's graph structure feature. It only uses a subset of an entity's neighbor node. Since the scale of the entity's graph decreases, EDEGE could be accelerated with GPU.

Graph convolutional network's input is a graph and output is every node's label. EDEGE enhances the node's feature according to its neighbor nodes. The process can be described as

(7) $$\begin{array}{c} H^{l+1}=\text{ReLu}\left(\tilde{A}H^{l}W^{l}\right), \end{array}$$

where $\tilde{A}$ is the normalized adjacency matrix with self-connected input graph and *H*^*l*^ and *W*^*l*^ are *l*-th layer of hidden state and weight.

Similar to graph convolutional network, EDEGE learns latent feature from the mention entity and its neighbor entity. Suppose that m*k* is the hidden state of neighbor entity *m*_*k*_; EDEGE expands them to *m*_*i*_'s current hidden state ${\tilde{h}}^{t}\in R^{\left(2qn+1\right)\times \text{d}t}$, so each row corresponds to the row of the adjacent matrix $\tilde{A}$. The subgraph convolutional is defined as

(8) $$\begin{array}{c} h^{t+1}=\text{ReLu}\left(\tilde{A}{\tilde{h}}^{t}W^{t}\right), \end{array}$$

where *W*_*t*_ ∈ *R*^d*t*×d*t*+1^ is the training parameter.

### 4.4. Proposed Algorithm

Algorithm 1 shows the entity disambiguation algorithm EDEGE based on a graph neural network. First, the random walk method is used to vectorize the triples in the knowledge graph, and then the vectorized triples are scored according to the scoring function. The size of the score finds out the ambiguous entities, then deletes the ambiguous entities from the triples, and puts them into the candidate triples. Finally, the candidate entities are sorted according to the cosine similarity, and the ambiguous entity with the highest rank is missing entities, thus achieving the goal of entity disambiguation. See Algorithm 1 for details.

## 5. Experimental Results and Analysis

### 5.1. Data Sets

We use standard data sets to validate the effectiveness of the proposed EDEGE.  Table 1 gives the statistical feature of the data sets used in these experiments. Among them, AIDA-CoNLL [13] is currently one of the largest artificially annotated entity disambiguation data sets. It is annotated based on CoNLL 2003 and contains 27,724 entities. We selected some of them as AIDA-B, which contains 4485 entity information. MSNBC [31] is a selection of 20 articles from different topics, with a total of 656 mentioned entities. AQUAINT [32] is a selection of 50 news articles from Xinhua News Agency, the New York Times, and the Associated Press, with a total of 727 linkable mentioned entities. ACE2004 [33] is a subset of the ACE2004 conference documents; it contains 257 mentioned entities within 35 articles through crowdsourcing.

### 5.2. Evaluation Metrics

We use Precision, Recall, and *F*1-measure to evaluate the proposed method, which is defined by formulas (9)–(11), respectively. Truth represents the number of ambiguous reference entities that exist in the test data set, and *result* represents the number of ambiguous entities that can be identified by the disambiguation method.

(9) $$\begin{array}{c} \text{precision}=\frac{\left|\text{truth}\cap \text{result}\right|}{\left|\text{result}\right|}, \end{array}$$

(10) $$\begin{array}{c} \text{recall}=\frac{\left|\text{truth}\cap \text{result}\right|}{\left|\text{truth}\right|}, \end{array}$$

(11) $$\begin{array}{c} F1=\frac{2*\text{precision}*\text{recall}}{\text{precision}+\text{recall}}. \end{array}$$

### 5.3. Baseline Methods

PBoH performs collective linking based on a probabilistic graph model, which counts Wikipedia statistical information on the cooccurrence of word and entity to perform entity disambiguation.

DoSeR is a collective disambiguation method utilizing the Personalized PageRank value on the entity graph of mentioned entities, and it employs the entity's graph feature for entity disambiguation.

NeuPL utilizes a deep neural network method to calculate the similarity match between the mentioned and target entities, and it uses the global semantic to solve the entity disambiguation task.

GNED is a graph neural network-based entity disambiguation method, which makes full use of the global semantic feature.

NCEL [34] applies graph convolutional network to integrate both local contextual features and global graph features for entity disambiguation.

E2ENEL [35] is a neural end-to-end entity linking system that unites, discovers, and links entities in a text document. It considers all probable spans as latent mentions and utilizes contextual similarity scores between entity candidates that are helpful for entity disambiguation.

BOOTLEG [36] is a self-supervised entity disambiguation system that utilizes reasoning patterns for disambiguation. It defines core reasoning patterns for disambiguation, creates a learning procedure to encourage the self-supervised model to learn the reasoning patterns, and encodes the patterns in a Transformer architecture.

### 5.4. Experimental Results

For a fair comparison, we employ the same parameters as baseline methods provided in [8, 9, 17, 25]. In EDEGE, the embedding size *d* = 300, the walk length of a node ***θ*** = 3, and the threshold for choosing neighbor node *α* = 0.68, which achieve the best results on the validation set.

Table 2 gives the microaverage *F*1 of EDEGE and the baseline method. The entity disambiguation model needs to link the entity to the candidate entity by their similarity; therefore, it needs to select an entity linking task to match the entity to the correct entity. All baseline algorithms are completely using local context features to perform entity linking. With entity links, mentions are associated with the entity with the highest match value. This paper could draw the following conclusions from the results. First, EDEGE acquires the highest performance on the data sets. Based on the average *F*1 value, its result is better than the GNED method which is based on graph neural networks. GNED uses entity graphs to construct the relationship between entities in the context. Although it considers the semantic information of entity relationships, it ignores the structural features between entities. EDEGE uses a graph neural network to capture entity-relationship semantic embedding and combines entity graph structure embedding for entity disambiguation. Without a collective matching scheme, EDEGE can achieve better results than GNED and NeuPL. Second, compared with NeuPL, DoSeR, and GNED, the performance of PBoH is lower than that of neural network-based methods. This is because using cooccurrence feature of word and entity cannot capture the semantic feature between entity relationships. On the contrary, using continuous representations embedded in entity relationships can improve the semantic matching of entity relationships. Third, in these 5 data sets, EDEGE performs slightly better on the AIDA-B data set. This is because this data set is manually labeled and the data quality is better. Compared with the PBoH based on the statistical method, the *F*1 value of the disambiguation result increased from 0.752 to 0.924.

The introduction of external knowledge will enhance the performance of entity disambiguation. This paper compares EDEGE with the baseline methods in the case of introducing DBpedia for entity disambiguation. The results of Precision, Recall, and *F*1 values are shown in Table 3. EDEGE achieves the best results in these data sets. On the AIDA-CoNLL data set, the *F*1 value of EDEGE is 7.2% higher than that of GNED, which ranks second. On the ACE2004 data set, the Precision, Recall, and *F*1 values of EDEGE are 9.2%, 7%, and 11.2% higher than the following method GNED, respectively. On average, EDEGE is better than GNED on the five data sets and the Precision, Recall, and *F*1 values are 7.7%, 7.5%, and 7.7% higher than those of GNED, respectively. Besides entity's semantic features and structure features, neural network-based methods could capture the latent features between entities. Therefore, these kinds of methods (such as NeuPL, GNED, NCEL, E2ENEL, and EDEGE) achieve the best performance among these methods. EDEGE achieves the best performance among neural network methods because it considers both entity's semantic feature and subgraph structure to do entity disambiguation task. Reason pattern-based methods (such as BOOTLEG) ranked as the second team and have good interpretability. Structure feature-based method DoSeR ranked as the third echelon because it only utilizes the entity's graph structure to disambiguate entities. The probability model-based method PBoH ranked last since it only uses the statistical feature to disambiguate entities.

**Figure 3:**
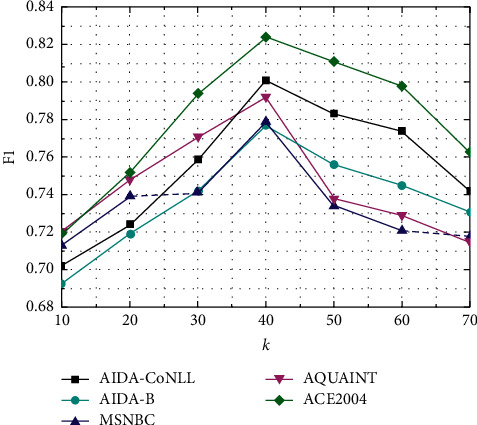
The influence of *k* to EDEGE on five data sets.

**Figure 4:**
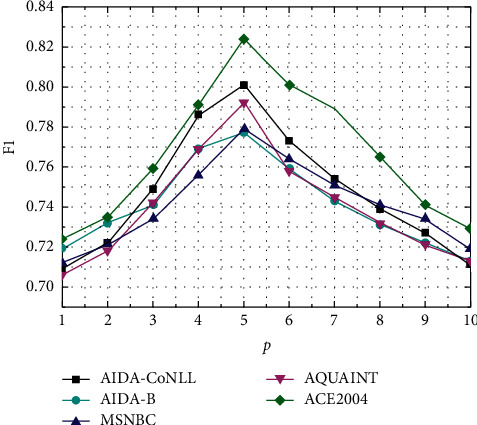
The influence of *p* to EDEGE on five data sets.

In Figures 3 and 4, this paper analyzes the influence of hyperparameters on the EDEGE method under five data sets. The parameters contain the number *k* of the most relevant words linked to an ambiguous entity to be disambiguated, the amount *p* of the most relevant entities to be disambiguated, and *ß* that assigns the semantic similarity of the entity relationship and the similarity of the subgraph structure.  Figure 3 indicates that when the amount *k* of the most relevant words of the entity is 40, EDEGE has achieved the best *F*1 value on the five data sets, of which the best results have been achieved on the AIDA-CoNLL data set. Its *F*1 value is 0.801. When *k* is larger than 40, the *F*1 value on five data sets drops because of entity sparseness in the knowledge graph.  Figure 4 indicates that the number of related entity *p* has a strong influence on the evaluation of the EDEGE method. When *p* increases, *F*1 gradually increases. When *p* is 5, *F*1 reaches the maximum value of 0.801, and then *F*1 gradually decreases as *p* increases. With the increasing of *p*, the noise entities will come to the entity's subgraph and cause poor performance. It is also found in the experiment that the influence of the parameters on all data sets is almost the same. Therefore, we can select parameters according to the experiment on the validation set as in the experiment.

## 6. Conclusions

This paper proposes an entity disambiguation model EDEGE for knowledge graph fusion, which fuses the entity-relationship vector similarity and entity subgraph-embedded representation similarity to solve knowledge graph entity conflict problems in the procedure of knowledge graph propagation. EDEGE uses the entity-relationship embedding to indicate the semantic relationship between entities, uses the structural features of the subgraph of the knowledge graph where the entity is located to represent the structure features of the entity and the surrounding entities, and fuses the two embedding representations through a balance factor to combine the fused vector as the input of the neural network, and the ambiguous entities are finally distinguished, to resolve entity conflict issue in the knowledge graph. A large number of experiments on public data sets indicate that EDEGE is better than the current entity disambiguation methods. Since the comprehensive consideration of the semantics of entity relations and the structural characteristics of subgraphs has a good performance in the entity disambiguation of the knowledge graph, it is proved that this idea is very effective in the embedded representation of the knowledge graph. Therefore, we will explore the effect of EDEGE in knowledge graph relationship conflict detection and error correction in future work.

## Figures and Tables

**Algorithm 1:**
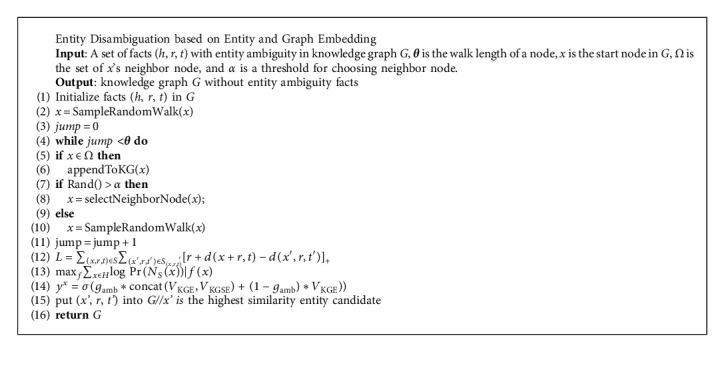
Entity disambiguation algorithm based on entity and graph embedding.

**Table 1:** Data set statistics information.

Data set	Number of mentions	Number of documents	Mentions per document
AIDA-CoNLL	27724	1393	19.9
AIDA-B	4485	231	19.4
MSNBC	656	20	32.8
AQUAINT	727	50	14.5
ACE2004	257	36	7.1

**Table 2:** *F*1 mean values of EDEGE and the baseline method on different data sets.

Method	AIDA-B	AIDA-CoNLL	MSNBC	AQUAINT	ACE2004	Average
PBoH	0.752	0.741	0.753	0.761	0.749	0.7512
DoSeR	0.895	0.885	0.725	0.876	0.883	0.8528
NeuPL	0.896	0.878	0.879	0.884	0.897	0.8868
GNED	0.899	0.893	0.882	0.887	0.898	0.8918
NCEL	0.878	0.879	0.889	0.87	0.89	0.8812
E2ENEL	0.892	0.866	0.873	0.882	0.894	0.8814
BOOTLEG	0.891	0.891	0.874	0.865	0.873	0.8788
EDEGE	**0.924**	**0.921**	**0.914**	**0.908**	**0.919**	**0.9172**

To distinguish the proposed EDEGE method from baseline methods, the maximum value is bold in each column.

**Table 3:** Disambiguation results of EDEGE and baseline methods with different knowledge graphs.

Data set	Knowledge graph	Method	*F*1	Precision	Recall
AIDA-CoNLL	DBpedia	PBoH	0.694	0.766	0.635
		DoSeR	0.708	0.791	0.641
		NeuPL	0.719	0.803	0.651
		GNED	0.729	0.809	0.663
		NCEL	0.717	0.806	0.656
		E2ENEL	0.704	0.805	0.658
		BOOTLEG	0.721	0.803	0.651
		EDEGE	**0.801**	**0.836**	**0.768**

AIDA-B	DBpedia	PBoH	0.668	0.698	0.641
		DoSeR	0.682	0.714	0.652
		NeuPL	0.701	0.737	0.669
		GNED	0.715	0.751	0.683
		NCEL	0.709	0.743	0.679
		E2ENEL	0.711	0.739	0.681
		BOOTLEG	0.708	0.744	0.676
		EDEGE	**0.777**	**0.812**	**0.744**

MSNBC	DBpedia	PBoH	0.677	0.705	0.652
		DoSeR	0.694	0.724	0.667
		NeuPL	0.705	0.698	0.713
		GNED	0.706	0.727	0.686
		NCEL	0.703	0.713	0.676
		E2ENEL	0.698	0.708	0.681
		BOOTLEG	0.702	0.715	0.679
		EDEGE	**0.779**	**0.823**	**0.739**

AQUAINT	DBpedia	PBoH	0.673	0.699	0.648
		DoSeR	0.686	0.718	0.657
		NeuPL	0.696	0.732	0.663
		GNED	0.706	0.728	0.686
		NCEL	0.693	0.724	0.679
		E2ENEL	0.702	0.713	0.681
		BOOTLEG	0.689	0.709	0.673
		EDEGE	**0.792**	**0.851**	**0.741**

ACE2004	DBpedia	PBoH	0.661	0.684	0.639
		DoSeR	0.680	0.705	0.657
		NeuPL	0.705	0.734	0.679
		GNED	0.732	0.762	0.705
		NCEL	0.724	0.753	0.701
		E2ENEL	0.718	0.741	0.698
		BOOTLEG	0.726	0.729	0.703
		EDEGE	**0.824**	**0.832**	**0.817**

To distinguish the proposed EDEGE method from baseline methods, the maximum value is bold in each column.

## Data Availability

The data used to support the findings of this study are included within the article.
